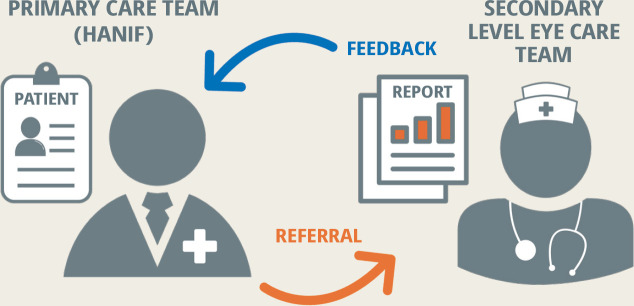# The role of secondary level eye care teams in engaging with and supporting primary eye care

**Published:** 2022-03-01

**Authors:** Daksha Patel

**Affiliations:** 1 Associate Professor of International Eye Health: International Centre for Eye Health, London School of Hygiene & Tropical Medicine, London, UK.

**Primary eye health care teams have the potential to drastically reduce the burden on secondary level eye care teams and to improve access and quality of care for patients – provided they receive the support they need.**

**Figure:**
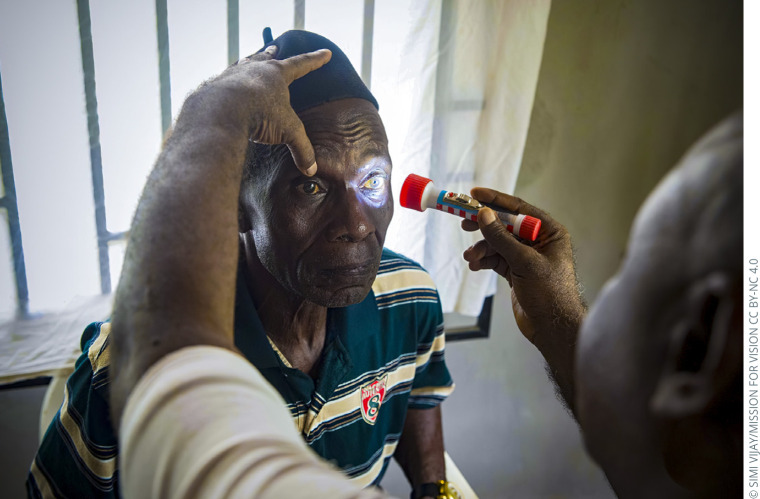
A simple torch examination can help primary care workers identify and refer patients who need eye care at secondary level. **NIGERIA**

In many countries, the team at the secondary level (often at district level) is made up of an ophthalmologist and optometrist, ophthalmic clinical officers and nurses. The secondary level is also often the first place where people with eye problems seek care, particularly in rural or remote settings where there are insufficient ophthalmic personnel. Ideally, a secondary eye unit has the resources needed to provide services for a population of 0.5 to 1 million people,^1^ where up 20-25% of the population are likely to have an eye condition needing services (including refractive error services).^2^

The secondary level team have an important role to play in supporting the primary care team – the staff members who provide primary eye health care (PEHC) in the community – even if this role is not formalised. Supporting local primary care teams to provide high quality eye care for simple eye conditions in the community can help to reduce the load on outpatient clinics in secondary and tertiary level facilities. This support ensures that high quality care is provided as close as possible to communities, saves patients’ time, and reduces costs – but it also allows eye specialists to focus on complex conditions and procedures. Supporting primary care workers to manage simple eye conditions can, therefore, improve efficiency and the quality of care for patients and the entire health system.

Most secondary eye units will have the resources needed to provide a range of surgical and medical eye care services. The delivery of these services are often enhanced by outreach activities to reach, for example, people with operable cataract in the catchment area of the hospital.2 These outreach activities should be planned and coordinated by working closely with primary care teams, who can inform the community of the date, time and location of the next outreach. This includes informing primary care teams when activities are cancelled.

Secondary eye units should inform primary care teams about the range of services they provide in the hospital and during outreach so that patients attending primary care facilities can be appropriately informed. This might include screening for diabetic retinopathy in clinics or outreach, or school eye health programmes in the area. In an ideal system, all patients would present to primary care with their eye problems; the primary care team members would be able to manage the vast majority of common conditions, e.g. conjunctivitis, and then refer on more complex conditions to secondary care. In some settings, particularly in Asia, some secondary units in the non-government sector provide teleconsultations for patients who are seen at the primary level.

## Providing feedback to primary care teams

One way secondary level eye care professionals can support primary eye health care is by providing feedback on referrals, indicating what role the primary care team member may have in ongoing care. Staff working in secondary hospitals also decide which patients need referral for tertiary care. If a patient has been referred to the secondary unit by a primary care team member and is then referred on for tertiary care, this should be also be communicated to them. Armed with this information, the primary care team member will be able to provide ongoing, patient centred care when the patient returns from the tertiary centre.

School eye health programmes run by secondary level eye units can also be enhanced by working with PEHC teams, as described elsewhere in this issue.

## Training

Secondary units provide invaluable in-service or refresher training for primary care providers. These opportunities allow for everyone to get to know each other, which builds trust, and the PEHC teams can see and understand first hand the services being provided. For example, as part of training, they may watch cataract surgery and be better equipped to answer patient questions at a community level. They could talk to patients who were happy after their cataract surgery, or who received spectacles, to understand the impact of treatment. They can be equipped to use health education and health promotion information and tools effectively.

## Tips for success

In most districts there are multiple primary health care clinics and teams for every secondary level eye care facility. **Good communication** is central to the partnership:

- Ensuring that detailed referral documentation between the two providers is used. This is important for health providers but also reassures patients who may not be familiar with specialist services and are likely to be anxious.
- Using feedback mechanisms for primary eye care providers and follow-up support which can be done through clear reporting forms and a range of m-Health (mobile health) options and alerts. Provide information about specific patients to ensure a continuum of care. For example, this might include the medication they need to use and the frequency, so that they can be counselled by primary level staff members. This is likely to improve adherence to treatment and improves capacity and outcomes within the health system.^3^
- Providing **scheduling information** on where and when outreach or other activities are planned, on a quarterly basis.

## Sharing evidence

Strengthening eye health services relies on collecting and using information at a local level, especially to identify who has been left behind. Secondary level teams often shoulder the responsibility for regularly planning services, reviewing and reporting on numbers of people who attend eye clinics and numbers that accept surgery. Therefore, sharing key information with primary eye care workers as health educators and case finders for cataract programmes can actively address some of the arising local issues, such as acceptance of cataract surgery by women.

The collaborative partnership between primary and secondary level eye care is constantly evolving, particularly as communications are improving using technology. Establishing formalised pathways between the two levels of services improves access and quality of care but also improves capacity within a health system.

**Referral and feedback: an example**

Hanif, a primary eye care provider, identified Miriam (78 years old) as having bilateral mature cataracts. He persuaded her family that she would benefit from surgery at the local district hospital. Using an agreed referral protocol, he provided the family with clear verbal and written instructions about when they should visit the hospital eye unit and what to expect. He also gave them the referral slip they would need to show when they arrived. Miriam and a companion travelled the 50 km to the hospital; she felt reassured as she knew what to expect.

A week after the cataract operation in the right eye, Hanif received feedback from the secondary team about the success of the operation, and when to visit Miriam. During his visit, Hanif saw that the eye was white, she had good vision, and no pain. He was also informed by the hospital that some changes had been made to her diabetes management while she was at the hospital.

Hanif was well informed and able to provide Miriam with the support and information she needed, and was able to report back to the secondary unit on her post-surgical recovery. Hanif felt reassured that he was managing the patient appropriately because he and the secondary level eye team maintained good communication throughout.

**Figure:**